# Clinicopathologic features and clinical outcomes of intravenous leiomyomatosis of the uterus: A case series

**DOI:** 10.1097/MD.0000000000024228

**Published:** 2021-01-08

**Authors:** Xiuzhang Yu, Jing Fu, Ting Cao, Liyan Huang, Mingrong Qie, Yunwei Ouyang

**Affiliations:** a Department of Obstetrics and Gynecology, West China Second Hospital, Sichuan University; b Key Laboratory of Birth Defects and Related Diseases of Women and Children, Sichuan University, Ministry of Education; c Department of Pathology, West China Second Hospital, Sichuan University, Chengdu, Sichuan, China.

**Keywords:** gonadotropin-releasing hormone agonists, intravenous leiomyomatosis, recurrence, smooth muscle tumors, uterus

## Abstract

**Rationale:**

Intravenous leiomyomatosis (IVL) is a rare and special type of smooth muscle tumor originating in the uterus. It is classified as a benign disease according to its histological features but shows the behavioral characteristics of a malignant tumor. It is easily misdiagnosed and recurrent. The purpose of this study was to retrospectively analyze clinicopathological data of 25 cases of IVL in order to enhance clinicians’ understanding of this rare disease.

**Patient concerns:**

We screened and identified 25 cases of IVL at our hospital from October 2013 to January 2020. Five patients had tumors.

**Diagnoses:**

The diagnosis in each case was pathologically confirmed after surgical treatment.

**Interventions:**

All patients were managed surgically. Although the surgical procedures were different, the surgical approach was geared towards achieving complete excision. Three patients received hormonal therapy with gonadotropinreleasing hormone agonists after surgery.

**Outcomes:**

We retrospectively reviewed all medical records and analyzed the clinicopathologic features and clinical outcomes of this disease as well as the correlations between the clinical features and risk of recurrence. Neither the symptoms nor the preoperative imaging results were suggestive of IVL in any of the cases. Except for two patients who were lost to follow-up, twenty-three patients who were followed up are still alive. Three patients experienced a recurrence.

**Lessons:**

The clinical manifestations and ultrasound images of IVL in the early stages are not typical; thus, IVL is easily misdiagnosed as uterine leiomyoma. Radiologists, pathologists, and surgeons should have a thorough understanding of IVL and a high index of vigilance for IVL in clinical practice. Surgery should always be aimed at achieving complete tumor excision. Patients with large lesions (≥7 cm) and lesions extending to the broad ligament may have an increased risk of recurrence. Early detection, diagnosis, and treatment are very important; once the diagnosis is confirmed, regular follow-ups are crucial.

## 1 Introduction

Intravenous leiomyomatosis (IVL) is a rare and special type of smooth muscle tumor that is classified as a benign disease according to its histological features but is malignant in terms of its behavior. Typically, IVL originates in the uterus, but it can extend along the venous system to the inferior vena cava, the right heart, and even the pulmonary artery.^[1]^ The clinical manifestations and ultrasound images of IVL in the early stages are not typical; thus, IVL is easily misdiagnosed or completely missed. As recurrence is frequent, it should be followed up more vigorously than ordinary uterine leiomyomas. IVL is relatively rare. The purpose of this study was to retrospectively analyze clinicopathological data of 25 cases of IVL to enhance clinicians’ understanding of this rare disease.

## 2 Materials and methods

### 2.1 Study subjects

We performed a retrospective review of 25 patients treated for IVL at our hospital from October 2013 to January 2020. The diagnosis of IVL was pathologically confirmed in all cases. This study was conducted with the approval of the hospital's Institutional Review Board. All patients have provided informed consent for publication of the case details.

All patients underwent surgical treatment. Surgical details were recorded by the surgeons during the operations. The postoperative pathological diagnoses were determined by 2 experienced pathologists according to the results of the hematoxylin and eosin staining and immunohistochemical staining of the tissue specimens. The medical records of each patient were reviewed. An attempt was made to follow up each patient.

### 2.2 Statistical analysis

The correlations between the clinical features and risk of recurrence were analyzed. Data pertaining to age were evaluated using the Mann–Whitney test, and differences in categorical data were evaluated using the Chi-squared test. A *P*-value below .05 was regarded as indicating a significant difference.

## 3 Results

The clinical and pathological features and follow-up data of the 25 patients are summarized in Table 1. Five of the patients had tumors. The ages of the patients ranged from 26 to 53 years (mean, 43.6 years); the median age was 45 years. Only 1 patient was postmenopausal. 20 patients (80%) had a history of gynecological or obstetric surgery, including myomectomy (6, 24%), cesarean section (11, 44%), and intrauterine device placement (4, 16%). The presenting symptoms were not specific and included uterine or pelvic masses, menorrhagia, menstrual disorders, hypogastralgia, frequent urination, and dysuria.

**Table 1:** Clinical and pathological features of 25 cases of intravenous leiomyomatosis.

Case	Age (yr)	Menopausal status	Previous surgical history	Presentation	Tumor size ≥ 7 cm	Macroscopic presence of intravascular fibroids	Lesions extenting to the broad ligament	Frozen pathology results prompted IVL	Ki-67 index <5%	Surgical strategy	Post-surgical therapy	Other pathology findings	Follow-up (mo)
1	48	N	CS	Uterine masses, frequent urination	N	Y	N	N	Y	TAH (LAP)	GnRH-a	Angioleiomyoma	5 mo NED
2	26	N	CS+myomectomy	Menorrhagia	N	N	N	N	Y	TAH	N	Leiomyoma with degeneration	11 mo NED
3	47	N	N	Uterine masses	N	N	RBL	N	Y	TAH	N	Leiomyoma	13 mo NED
4^∗^	48	N	CS	Hypogastralgia	Y (10 cm)	N	BBL	N	Y	TAH (LAP)	N	Angioleiomyoma	US found a 4 cm pelvic mass in 1 mo after surgery and it is still there (13 mo after surgery).
5	42	N	CS	Menorrhagia	Y (10 cm)	N	N	N	Y	TAH	N	Angioleiomyoma	14 mo NED
6	48	N	Myomectomy	Hypogastralgia	Y (8 cm)	N	N	N	N (approximately 10%)	TAH (LAP)	N	Cellular leiomyoma	15 mo NED
7	49	N	CS	Uterine masses	N	N	N	N	Y	TAH and BSO (LAP)	N	Leiomyoma with degeneration	17 mo NED
8	43	N	Myomectomy	Menorrhagia	N	Y	N	N	Y	TAH and BSO (LAP)	N	Leiomyoma and adenomyosis	18 mo NED
9	45	N	Myomectomy	A pelvic mass	N	N	LBL	N	Y	TAH (LAP)	N	Leiomyoma with degeneration	23 mo NED
10	45	N	N	Menstrual disorders	N	Y	RBL	N	Y	TAH and LSO (LAP)	N	Adenomyosis	29 mo NED
11	37	N	CS	Menorrhagia	Y (10+ cm)	N	N	Y	Y	Myomectomy	GnRH-a	Leiomyoma	39 mo NED
12	50	N	N	Menorrhagia	N	N	N	Y	Y	TAH and BSO (LAP)	N	Leiomyoma	Lost to follow-up
13	44	N	CS	A pelvic mass	Y (15 cm)	N	LBL	N (cellular leiomyoma (mfc0–1/40 mpf))	N (approximately 10%)	Myomectomy (LAP)	N	Angioleiomyoma and cellular leiomyoma	49 mo NED
14^∗^	39	N	Myomectomy	A pelvic mass, hypogastralgia	Y (7 cm)	Y	RBL	Y	Y	Modified radical hysterectomy	GnRH-a	Endometriosis	US found a 2 cm pelvic mass in 13 mo after surgery. Treatment with GnRH-a. CT found a 5 cm pelvic mass 43 mo after surgery.
15	39	N	IUD	Pelvic masses, dysuria	Y (8 cm)	Y	N	N	Y	TAH	N	Leiomyoma	57 mo NED
16^∗^	39	N	N	Menorrhagia, hypogastralgia	Y (7 cm)	N	RBL	N	Y	Myomectomy	N	Angioleiomyoma with degeneration	US found a 1+ cm uterine myoma in 36 mo after surgery and it increases to 3 cm now (58 mo after surgery).
17	53	Y	N	Uterine masses	N	N	N	N	Y	TAH and BSO	N	Leiomyoma	77 mo NED
18	45	N	CS	Uterine masses	Y (10 cm)	N	RBL	Y	Y	Subtotal hysterectomy (LAP)	N	Leiomyoma with degeneration	78 mo NED
19	45	N	IUD	Uterine masses	Y (9 cm)	N	RBL	Y	Y	TAH	N	Leiomyoma and adenomyosis	Lost to follow-up
20	41	N	IUD	Menorrhagia	N	N	N	N	Y	TAH	N	Angioleiomyoma with degeneration	80 mo NED
21^†^	48	N	CS	Menorrhagia, diagnostic curettage abnormalities	N	N	N	N	Y	TAH and BSO (LAP)	N	Adenomyosis, endometrial well-differentiated endometrioid adenocarcinoma	12 mo NED
22^†^	44	N	Myomectomy	Uterine masses	Y (7 cm)	Y	BBL	N	Y	TAH+BSO	Chemotherapy (IAP)	Leiomyoma, uterine adenosarcoma	19 mo NED
23^†^	46	N	CS	Frequent urination, cervical biopsy abnormalities	Y (10 cm)	Y	N	N	Y	Radical hysterectomy+BSO	N	Cervical moderately differentiated squamous cell carcinoma, leiomyoma	23 mo NED
24^†^	50	N	IUD	Pelvic masses, hypogastralgia	N	N	N	N	Y	TAH and BSO	Chemotherapy (IAP)	Endometrial adenosarcoma, leiomyoma	53 mo NED
25^†^	29	N	CS	Cervical biopsy abnormalities	N	N	N	N	Y	Radical hysterectomy+BSO	Radiotherapy	Cervical mid-low differentiated mucinous adenocarcinoma, leiomyoma	73 mo NED

**Figure 1:**
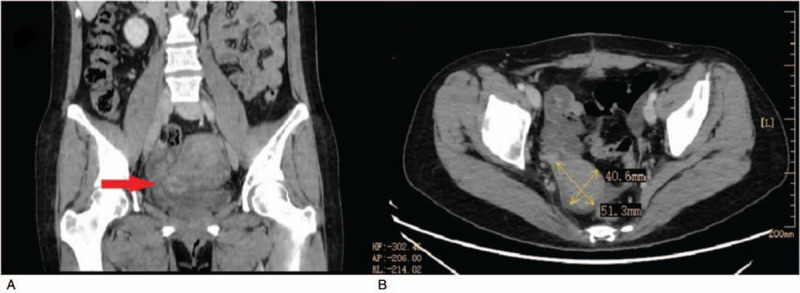
computed tomography scans of case 14. (A) preoperation: a mass of slightly higher density shadows was seen on the right side of the uterus, and the contrast-enhanced scan showed nodular and ring enhancements (arrow). (B) Forty-three months after surgery: A slightly hypodense solid soft masses, which was slightly enhanced in enhancement scan computed tomography scans, was found in the right posterior part of the pelvic cavity.

All patients underwent a preoperative ultrasound examination, and the diagnoses included uterine myoma, adenomyosis, or unspecified space-occupying lesions. Almost all ultrasound scans suggested that blood flow signals were detected around and/or within the mass. Some patients were examined using computed tomography (CT), but all were diagnosed as having uterine fibroids or pelvic masses instead of IVL (Fig. 1). Of the 20 patients without tumors, 14 were tested for tumor markers; 8 of them had normal tumor marker test results, and the remaining 6 had slightly elevated levels of alpha-fetoprotein, cancer antigen 125, or cancer antigen 19-9.

In all cases, the surgical approach was geared towards achieving complete excision. Five patients with tumors were treated according to the standard protocols. Of the remaining 20 patients, 3 underwent myomectomy, 10 total abdominal hysterectomy (TAH), 1 modified radical hysterectomy, 1 subtotal hysterectomy, 4 TAH and bilateral salpingo-oophorectomy (BSO), and 1 TAH and left salpingo-oophorectomy. Half the number of procedures were laparoscopic surgeries and the other half were laparotomies. Three patients received hormonal therapy with gonadotropin-releasing hormone agonists (GnRH-a) after surgery (Table 1).

**Figure 2:**
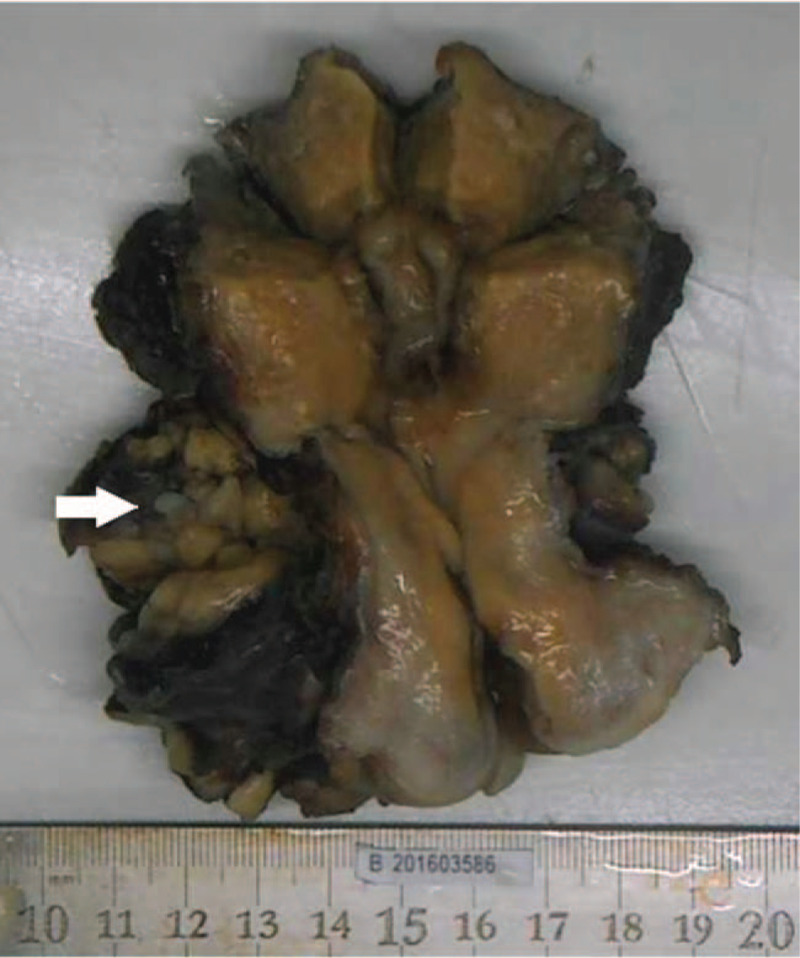
Gross images of case 14. Lesions extending to the broad ligament, and intravenous leiomyomatosis were visible in parauterine vessels.

All patients had enlarged uteruses. The lesions were larger than 7 cm in 12 cases, fibroid-like or cord-like tissues were found in the vessels in 7 cases, and lesions extending to the broad ligament were found in 10 cases (Table 1) (Fig. 2). In case 8, beaded white myoma-like nodules were found in the vascular section of the funnel, inherent ligament of the ovary, and uterine vein. In case 10, worm-like plugs were found within the ovarian vein. No intravenous leiomyomas were detected in the iliac veins or inferior vena cava. The intraoperative frozen-section pathology results in 4 patients prompted the diagnosis of IVL; the other patients were mainly diagnosed as having leiomyomas or angioleiomyomas.

**Figure 3:**
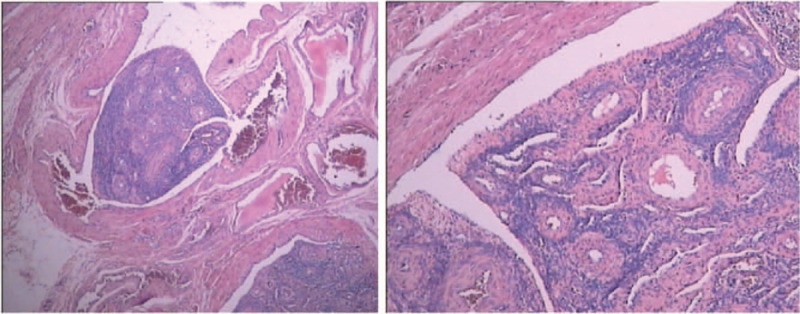
Typical histopathology of intravenous leiomyomatosis (hematoxylin & eosin staining).

All cases were characterized by the proliferation of benign smooth muscle within the vessels on microscopic examination (Fig. 3). Variable degrees of vascularity and hyalinization were present in some lesions. The histological evaluation showed positive results for caldesmon, desmin, estrogen receptor (ER), and progesterone receptor (PR) on immunohistochemical staining. In most cases, there was positive CD34 expression and negative or focal positive CD10 expression. The specimens had a Ki-67 index ranging from <1% to approximately 10%. The postoperative pathological examination did not reveal IVL as the sole diagnosis, and the concurrent gynecological disorders included angioleiomyoma, leiomyoma (cellular leiomyoma), adenomyosis, endometriosis, endometrial adenocarcinoma, endometrial adenosarcoma, uterine adenosarcoma, cervical carcinoma, and cervical adenocarcinoma.

Two patients were lost to follow-up. Twenty-three patients who were followed up are still alive. The follow-up duration was 5 to 80 months with a mean of 36 months and a median time of 49 months. None of the 5 patients with tumors experienced a recurrence of either the tumor or of IVL. Of the remaining 18 patients, a 2 cm pelvic mass was revealed on ultrasound 13 months after surgery in a patient (case 14). This patient had undergone a modified radical hysterectomy and received GnRH-a treatment after surgery. She refused to undergo any more surgeries and is still under GnRH-a treatment. However, at present the mass has increased in size to 5 cm. Another patient (case 4) who had undergone TAH underwent an ultrasound examination at a local hospital 1 month after surgery, which suggested a pelvic space occupying lesion of 4 cm. However, the patient did not seek further treatment because of the 2019 novel coronavirus disease outbreak. In another patient (case 16) who underwent myomectomy, a uterine fibroid >1 cm was found on ultrasound 3 years after the operation, and the fibroid gradually increased in size to 3 cm, but the patient did not consider reoperation. No recidivate or metastatic masses were detected in the remaining patients. The correlation analysis between the clinical features and risk of recurrence is summarized in Table 2. The risk of recurrence was not influenced by factors such as age, menopausal status, previous surgical history, macroscopic presence of intravascular fibroids, the Ki-67 index, surgical strategy, or post-surgery GnRH-a hormonal therapy. Lesions extending to the broad ligament were more prone to recur (*P* = .021). If the tumor size was ≥7 cm, the risk of recurrence was also high, but the efficiency of the test was insufficient (*P* = .052).

**Table 2:** The correlation analysis between the clinical features and risk of recurrence.

	Non-recurrence N (%)	Recurrence N (%)	*P*^a^
Total	20	3	
Age, yr (range)	45.0 (26.0,53.0)	39.0 (39.0, 48.0)	.492
Menopausal status			
YES	1 (5.0)	0 (0.0)	.692
NO	19 (95.0)	3 (100.0)	
Previous surgical history			
YES	17 (85.0)	2 (66.7)	.435
NO	3 (15.0)	1 (33.3)	
Tumor size			
≥ 7cm	8 (40.0)	3 (100.0)	.052
<7cm	12 (60.0)	0 (0.0)	
Macroscopic presence of intravascular fibroids			
YES	6 (30.0)	1 (33.3)	.907
NO	14 (70.0)	2 (66.7)	
Lesions extenting to the broad ligament			
YES	6 (30.0)	3 (100.0)	.021^∗^
NO	14 (70.0)	0 (0.0)	
Ki-67 index<5%			
YES	18 (90.0)	3 (100.0)	.567
NO	2 (10.0)	0 (0.0)	
Surgical strategy			
TAH/TAH and LSO	10 (50.0)	2 (66.7)	.297
TAH and BSO	8 (40.0)	0 (0.0)	
myomectomy	2 (10.0)	1 (33.3)	
Post-surgical therapy			
GnRH-a	2 (10.0)	1 (33.3)	.263
Non GnRH-a	18 (90.0)	2 (66.7)	

## 4 Discussion

In this study, we found that the clinical manifestations and ultrasound images of IVL in the early stages are not typical; thus, IVL is easily misdiagnosed as uterine leiomyoma. Radiologists, pathologists, and surgeons should have a thorough understanding of IVL in clinical practice. Surgery should always be aimed at complete tumor excision. Patients with large lesions (≥7 cm) and lesions extending to the broad ligament may have an increased risk of recurrence. IVL was first described in 1896 as a tumor with the potential to infiltrate the veins.^[2]^ IVL is rare; Du et al reported that IVL comprised approximately 0.097% of all genital smooth muscle tumor cases at their hospital.^[3]^ The related literature is limited, and most studies have described advanced disease with right atrium involvement. If handled properly, this situation may be avoided. Herein, we described our experience regarding the timely diagnosis and treatment of 25 cases of early-stage IVL.

Histologically, IVL presents as a benign leiomyoma; the disease may be confined to the uterine vein, but it may also invade the pelvic vein, inferior vena cava, renal vein, pulmonary artery, etc, and may cause distant metastasis to the cardiac, pulmonary. IVL invades the systemic venous circulation mainly via the uterine or ovarian vein.^[4]^ The pathogenesis of IVL remains to be elucidated. There are 2 principal theories:

(1) that the smooth muscle cells in the blood vessel wall grow into the lumen,
(2) that the leiomyoma invades the uterus or parauterine veins.^[5]^

It was reported that the intravascular tumor cells always exhibited ER and PR positivity, while the endothelial and subendothelial cells expressed none or scant or weak positivity; thus, it is likely that IVL originates from uterine leiomyomas.^[6]^ IVL is more common in premenopausal women. Most patients with IVL have uterine fibroids or a history of myomectomy/hysterectomy.^[7]^ Elevated estrogen levels, venous blood stasis, and local injuries such as a history of uterine surgery may be important factors for the onset of the disease.^[8]^ Our study showed that cesarean section and intrauterine device placement may also be related to IVL, a finding that has rarely been reported before. Moreover, our study included 5 patients with tumors, which is rare in previous reports. As mentioned earlier, IVL is a benign disease that is malignant in behavior. Studies have shown that the expression profile of IVL is similar to that of leiomyosarcomas.^[9]^ The relationship between IVL and other tumor-producing diseases is worth studying further.

Clinically, uterine IVL can be divided into 4 stages.^[10]^ Tumors invading the uterine vein and limited to the pelvis are considered stage I. Tumors extending to the abdominal cavity but without involvement of the renal vein are considered stage II. Tumors invading the renal vein and inferior vena cava and extending further up to the right atrium but without reaching the pulmonary arteries are considered stage III. Tumors reaching the pulmonary arteries and/or metastasizing to the lungs are considered stage IV. Consistent with the findings of our study, almost all studies showed that the clinical manifestations of stage I patients lacked specificity. It was similar to uterine leiomyomas, manifesting as menorrhagia, hypogastralgia, masses in the pelvis, and compression symptoms caused by the masses.^[11]^ If the lesion involves the inferior vena cava, heart, or pulmonary artery, it can cause chest tightness, shortness of breath, lower limb swelling, recurrent syncope, postural hypotension, cardiac insufficiency, pulmonary embolism, Budd-Chiari syndrome, shock, or even sudden death due to mechanical obstruction.^[7,12]^ The diagnostic value of preoperative imaging examination in IVL may be limited. In our study, the preoperative imaging results did not suggest IVL in any of the cases. The performance of ultrasound in IVL is similar to that seen in cases of uterine fibroids, and color Doppler flow imaging always reveals abundant blood flow.^[13]^ When IVL infiltrates the parauterine blood vessels, it is often misdiagnosed as broad ligament fibroids or ovarian tumors.^[14]^ It is difficult to distinguish IVL from an ordinary leiomyoma even via CT or magnetic resonance imaging, but these techniques aid in revealing the relationship between venous masses and pelvic masses, which is helpful in determining the extent of uterine IVL.^[15–17]^ CT angiography can display the path of tumor extension, which is of great significance in advanced cases to enable appropriate surgical planning.^[18]^

The general pathology of IVL shows the following characteristics: nodule-like masses of smooth muscle cells grow in the venous lumen. The uterus is often enlarged, mostly due to uterine fibroids, and tumors can be seen in the uterine wall, parauterine ligament, uterine vein, and ovarian vein, appearing as cord-like, worm-like, or lobulated structures. The cut surface is Grayish-White or Pinkish-White. There is a gap between the tumor tissue and the blood vessel wall, which is easy to separate and peel off. At this time, the surgeon should be vigilant. The pathologist should use the correct method for sampling and embedding. The specimen must include the tumor and the surrounding uterine smooth muscle tissue, and the normal muscle layer should be cut parallel to the tumor to avoid tumor retraction or protrusion from the vein, resulting in a missed diagnosis.^[7,19]^ Microscopically, the tumors are located in the venous lumina lined with endothelial cells. The spindle-shaped smooth muscle cells are relatively consistent, with no or occasional mitotic figures. Occasionally, mucoid degeneration may be seen, especially in younger patients. Specific immunohistochemical markers include vimentin, desmin, smooth muscle actin, and PR and ER positivity. In most cases, there is negative or focal positive CD10 expression and positive CD34 expression, indicating that the tumor thrombus originates from smooth muscle cells rather than endometrial stromal cells or vascular endothelial cells. The Ki-67 index is usually less than 5%.^[6,10,19,20]^ This is consistent with our findings, but we observed that the Ki-67 levels of the 2 non-tumor cases were approximately 8% and 10%. The main differential diagnoses include uterine vascular leiomyoma, low-grade malignant endometrial stromal sarcoma, uterine leiomyosarcoma with vascular invasion, and cotyledonoid dissecting leiomyoma (Sternberg tumor).

Surgical resection is the main treatment for IVL. In principle, hysterectomy is recommended for most patients. For patients who are menopausal or nearing menopause (≥40 years) or have extrauterine vascular infiltration, bilateral oophorectomy may be recommended.^[3,12]^ Myomectomy is only suitable for young women with fertility needs. Complete removal of the lesion is crucial to obtain a favorable prognosis. Some scholars propose that women who do not have fertility requirements should be recommended to undergo unilateral salpingo-oophorectomy even if they are young, but others believe that neither ovarian resection nor postoperative hormonal therapy is associated with recurrence.^[3,12]^ Retaining the bilateral ovaries may be considered in young patients. When the lesion involves the renal vein, inferior vena cava, or the heart and blood vessels of the lungs, multidisciplinary surgery is required.^[10]^ As ER and PR are present in venous leiomyoma cells, adjuvant therapy with GnRH-a or other anti-estrogen drugs may be effective before or after surgery or even in inoperable cases. However, whether this strategy can reduce recurrence remains controversial.^[15,21–24]^ Aromatase inhibitors administered orally after surgery have also been described in some studies.^[10,25]^ IVL is easily recurrent even after surgery. The literature reports that the recurrence rate is approximately 16.6 to 30%.^[3,21,26]^ The probability of tumor recurrence is closely related to the degree of tumor resection.^[15]^ The symptoms of recurrence are usually a pelvic mass or signs of venous obstruction. A younger age, the size of the initial tumor, and large vein involvement may be high-risk factors for recurrence.^[7,12]^ Therefore, long-term follow-up after surgery should be performed to detect recurring lesions early and observe the dynamic changes in residual lesions. Follow-up should include a systematic examination, especially of the reproductive system, regular ultrasound examinations of the cardiovascular system, and pelvic and enhanced CT or magnetic resonance imaging imaging if necessary. However, there is no consensus on the periodicity of the follow-up. Some scholars suggest a baseline CT scan 6 months after surgery and subsequently, every 2 to 5 years, but others recommend a more closely spaced surveillance regime (every 3–6 months).^[7,27]^ For IVL recurrence, surgery is still the most effective treatment if conditions permit. In our study, the recurrence rate of IVL was 13% (3/23), which is lower than the rate described in previous reports. This may be because all patients in our study had early-stage disease; therefore, early diagnosis and treatment are of great significance in disease prognosis. The ages of the 3 patients who showed a recurrence were 48, 39, and 39 years. The treatment modalities were THA, modified radical hysterectomy with GnRH-a, and myomectomy, but none of the patients were willing to undergo BSO. All 3 patients had tumors ≥7 cm and lesions extending to the broad ligament as the common features. Cord-like tissue was observed on both sides of the parauterine vessels of a 39-year-old patient during the operation, in this case, despite prolonged postoperative GnRH-a therapy, a pelvic mass was found, and the lesion continued to increase in size. Unfortunately, the 3 patients who showed a recurrence refused further surgery. The correlation analysis between the clinical features and recurrence risk showed that patients with large lesions (≥7 cm) and lesions extending to the broad ligament may have an increased risk of recurrence. For young patients like these (aged approximately 40 years), BSO and post-surgery GnRH-a hormonal therapy may be considered, but whether these methods can reduce the risk of recurrence is a matter of further research.

The limitation of this study is its small sample size as only 25 patients were included. However, this is also due to the rarity of IVL. And this is a retrospective study, the information is limited. It is hoped that clinical institutions in other regions and countries will participate in IVL research in the future. In addition, we, too, intend to maintain a long-term follow-up of these patients to further understand the long-term prognosis of IVL.

## 5 Conclusion

In summary, radiologists, pathologists, and surgeons should have a good understanding of IVL and a high index of vigilance for IVL in clinical practice. If ultrasound examination reveals tubular or fissure-like echoless areas in the tumor, and color Doppler flow imaging identifies venous blood flow signals in fissure-like echoes, IVL should be considered. Careful exploration should be performed during the operation. If the veins are found to be cord-like, especially on the sides of the lower uterine segment, the broad ligament, parauterine tissue, or the area of attachment, surgeons should be vigilant and should try to remove the lesion completely, which is essential to reduce recurrence. Pathologists should perform careful sampling and tissue selection to include the tumor and surrounding uterine smooth muscle. Patients with large lesions (≥7 cm) and lesions extending to the broad ligament may have an increased risk of recurrence. Early detection, diagnosis, and treatment are very important; once the diagnosis is confirmed, regular, long-term follow-ups are crucial.

## Author contributions

**Conceptualization:** Yunwei Ouyang.

**Data curation:** Xiuzhang Yu, Ting Cao.

**Formal analysis:** Xiuzhang Yu, Ting Cao, Liyan Huang.

**Funding acquisition:** Mingrong Qie, Yunwei Ouyang.

**Investigation:** Xiuzhang Yu.

**Methodology:** Xiuzhang Yu, Jing Fu.

**Project administration:** Jing Fu.

**Resources:** Liyan Huang.

**Supervision:** Yunwei Ouyang, Mingrong Qie.

**Writing – original draft:** Xiuzhang Yu.

**Writing – review & editing:** Xiuzhang Yu.
